# Effects of ER-resident and secreted AGR2 on cell proliferation, migration, invasion, and survival in PANC-1 pancreatic cancer cells

**DOI:** 10.1186/s12885-020-07743-y

**Published:** 2021-01-07

**Authors:** Xian Hong, Zhi-Xuan Li, Jie Hou, Hui-Yu Zhang, Chun-Yan Zhang, Jian Zhang, He Sun, Li-Hong Pang, Tao Wang, Zhi-Hui Deng

**Affiliations:** 1 Laboratory of Protein Structure and Function, Institute of Medicine and Pharmacy, Qiqihar Medical University, Qiqihar, 161006 Heilongjiang China; 2 Department of gastroenterology, Third Affiliated Hospital, Qiqihar Medical University, Qiqihar, 161000 Heilongjiang China; 3 Department of gynaecology and obstetrics, Second Affiliated Hospital, Qiqihar Medical University, Qiqihar, 161001 Heilongjiang China

**Keywords:** AGR2, Drug sensitivity, ER stress, Pancreatic cancer

## Abstract

**Background:**

Anterior gradient-2 (AGR2) is a proto-oncogene involved in tumorigenesis and cancer progression. AGR2, predominantly localized in the endoplasmic reticulum (ER), is also a secreted protein detected in the extracellular compartment in multiple cancers. However, the biological functions of intracellular and extracellular AGR2 remain to be elucidated.

**Methods:**

Based on the biochemical structure of AGR2 protein, PANC-1 pancreatic cancer cells stably expressing ER-resident or secreted AGR2 were generated by a lentivirus-mediated stable overexpression system. The capacities of cell proliferation, migration, invasion and survival were assessed in PANC-1 stable cells. Moreover, EGFR expression and activation were determined to explore the possible mechanism of AGR2 roles in pancreatic cancer tumorigenesis.

**Results:**

It was discovered that secreted AGR2, but not ER-resident AGR2, promotes cell proliferation, migration and invasion of PANC-1 cells. Moreover, the data indicated that both the ER-resident and the secreted AGR2 enhance the survival capacity of PANC-1 cells after tunicamycin-induced ER stress and gemcitabine treatment. However, EGFR expression and activation were not found to be involved in AGR2-dependent oncogenic phenotypes in PANC-1 cells.

**Conclusions:**

Secreted AGR2 is predominantly involved in cell proliferation, migration and invasion in PANC-1 pancreatic cancer cells. Both secreted and ER-resident AGR2 contribute to the survival of PANC-1 cells under the challenging conditions. These findings provide insight into how different localizations of AGR2 have contributed to pancreatic cancer growth, metastasis, and drug sensitivity.

**Supplementary Information:**

The online version contains supplementary material available at 10.1186/s12885-020-07743-y.

## Background

Pancreatic cancer is an extremely aggressive solid malignant tumor. Its 5-year survival rate after diagnosis is less than 5% [1]. Currently, surgical resection is considered the only effective method for long-term survival for patients. Although radical resection is possible in 15 to 20% of cases, the 5-year survival rate of those patients is less than 10% [2]. Due to its insidious onset and the rapid progression of the disease, most patients are diagnosed with advanced or distant metastasis and often lose good opportunities for surgery. It is urgent to find new strategies for early detection and effective therapeutic intervention.

The human anterior gradient-2 (AGR2), an ortholog of the *Xenopus laevis* cement gland protein, resides in the endoplasmic reticulum (ER) and is a member of the protein disulfide isomerase (PDI) [3]. Because AGR2 has a strong link with carcinogenesis and tumor dissemination, it has been widely recognized as a proto-oncogene [4–12]. Overexpression of AGR2 has been reported in multiple solid human tumors, including breast, prostate, ovarian, lung, esophageal, gastric, colorectal and pancreatic cancers, suggesting it could be a unique biomarker in these tumors [4, 13–19]. Accumulating evidence suggests that AGR2 is a secretory molecule; its protein levels are found to be elevated in blood samples in several types of cancer patients [8, 15, 16, 20, 21] and the urine of prostate cancer patients [22]. Moreover, it is associated with poor prognosis in some solid tumors [23–26], and has been detected in circulating tumor cells and cancer stem cells [27–29]. Therefore, AGR2 may be a useful biomarker for diagnosis and prognosis of the cancers. Moreover, AGR2 is also a potential drug target. In vitro and in vivo studies showed that AGR2-targeting monoclonal antibody, selective peptide, and micro RNA can inhibit cancer cell growth and migration and enhance drug sensitivity [30–32].

It has been demonstrated that AGR2 levels are elevated in a majority of pancreatic cancer cell lines, pancreatic intraepithelial neoplastic lesions, and pancreatic cancer lesions [4, 33]. Previous studies show that intracellular AGR2 is predominantly localized in the ER of pancreatic cancer cells [7, 33] and is induced by ER stress [28]. It’s also involved in pancreatic cancer initiation [28]. Furthermore, AGR2 has been detected in conditional media culturing several pancreatic cancer cell lines, indicating that it is secreted [4]. However, the functional roles of extracellular and intracellular AGR2 in pancreatic cancer cells remain to be elucidated. Based on the molecular characteristics of AGR2, PANC-1 cell lines with stable expressions of ER-resident and secreted AGR2 were generated using lentiviral constructs. The aim of the following study is to explore the contribution and possible mechanism of intracellular and extracellular AGR2 to cell proliferation, migration, invasion, and survival in PANC-1 pancreatic cancer cells.

## Methods

### Cell culture and treatment

Human pancreatic adenocarcinoma PANC-1 and HEK 293 T cells were purchased from the Cell Bank of Chinese Academy of Sciences (Shanghai, China). Cells were cultured in Dulbecco’s Modified Eagle Media (DMEM), which contained 10% fetal bovine serum (FBS) in a humidified incubator with 5% CO_2_ at 37 °C. PANC-1 stable cells were plated, and then incubated with indicated concentration of tunicamycin (Aladdin, T101151) or 20 μM gemcitabine (Aladdin, G12018) for the indicated periods of time.

### Production of Lentiviral particles and generation of stable cell lines

The human wild type (WT) and deletion mutant lacking a C-terminal KTEL motif (△KTEL) of AGR2 cDNA (NCBI access number NM_006408) were amplified by polymerase chain reaction (PCR), then cloned into a modified pLenti6.3/V5-TOPO empty vector (pLenti6.3 MCS). This modified lentiviral expression vector does not contain V5-epitope anymore, and is a gift from Jin-San Zhang (Mayo Clinic, Rochester MN). To avoid mislocalization, no tag was added at both the N-terminal and C-terminal of the AGR2. All constructs were confirmed by DNA sequencing. HEK 293 T cells were used to produce lentiviral particles, and were cotransfected with pLenti6.3-AGR2-WT, -AGR2-△KTEL, or an empty vector with psPAX2 (addgene) and pMD2.G (addgene) using polyethyleneimine (PEI, Polysciences, 23966). PANC-1 cells were transduced with collected lentiviral particles, and were selected using 20 μg/mL Blasticidin (Thermo Fisher, A1113903) for 1 week.

### Reverse transcription-PCR (RT-PCR)

The total RNA was extracted from the cells using Total RNA purification kit (Genemark, TR01), and One Scrip cDNA synthesis kit (Abm, G233) was used for cDNA synthesis, according to the manufacturer’s instructions. The PCR program was under the following conditions: 94 °C for 3 min, 30 cycles of 30s at 94 °C, 30s at 55 °C, 70s at 72 °C, and 5 min at 72 °C for the final PCR product extension. Amplified products were separated on 1.5% agarose gels and visualized by ethidium bromide. GAPDH was used as a loading control for the PCR reactions. The primer sequences used for PCR are as follows: AGR2 (forward) 5’atggagaaaattccagtgtc3’, (reverse) 5’ctacagcaacttgagagctttcttc; GAPDH (forward) 5’aatgaaggggtcattgatgg3’, and (reverse) 5’aaggtgaaggtcggagtcaa3’.

### Purification of recombinant AGR2 proteins

GST-tagged AGR2-WT and AGR2-△KTEL were generated by subcloning into pGEX-6p-1 (GE Healthcare, 27-4597-01). Fusion proteins were purified from *E. coli* BL21 (Life Technologies, 44-0048) using glutathione immobilized Magnetic Agarose Beads (Sangon Biotech, C650031). Recombinant proteins were analyzed by SDS-PAGE and stained with Coomassie.

### Western blotting

Whole cell lysates were prepared in NP-40 lysis buffer supplemented with protease inhibitors. For the detection of secretory AGR2, conditioned media from cell lines were collected and concentrated 10× using Centricon YM-3 filter devices (Millipore Corporation). DSP-mediated chemical crosslinking for non-reducing SDS-PAGE was performed as previously described [34]. The proteins were separated by 12% SDS-PAGE and transferred to a PVDF membrane. The membrane was blocked with 4% bovine serum albumin (BSA) in PBS and probed with the appropriate antibodies. The primary antibodies used were as follows: anti-AGR2 described previously [14], α-tubulin (Santa Cruz, sc-8035), cleaved Caspase-3 (Cell Signaling Technology, 9664), Caspase-3 (Cell Signaling Technology, 9662), CHOP (Proteintech Group, 15204-1), PARP1 (Proteintech Group, 66520-1), EGFR (Proteintech Group, 18986-1), phosphorylated EGFR (Abcam, ab40815) and GAPDH (Proteintech Group, 60004-1). The protein signals were detected using Amersham Imager 680 (General Electric Company, USA).

### Immunofluorescent staining

Cells grown on coverslips were fixed with 4% formaldehyde, permeabilized with 0.15% Triton X-100, and blocked with 5% BSA. The coverslips then were incubated with rabbit anti-AGR2 (1:500, gifted from Dr. Jin-San Zhang [14]) and mouse monoclonal anti-calnexin (1:250, Thermo Fisher, MA3-027). The secondary antibodies were Alexa Fluor 568-conjugated goat anti-rabbit-IgG (1:600, Thermo Fisher, A-11036) and Alexa Fluor 488-conjugated goat anti-mouse IgG (1:400, Thermo Fisher, A-11001). DNA was stained with Hoechst 33342 (Thermo Fisher, H1399). Images were acquired by confocal microscopy (Cael Zeiss, LSM-710, Germany).

### Wound-healing assay

The cells (0.5-1 × 10^6^) were seeded in 6-wells plates (Corning Incorporated, USA), grown to about 95% confluence, and starved in a serum-free medium overnight. Next, the wound was made in a cell monolayer using the pipette tips in the middle of each well. Then, the cells were cultured for 24 h. Cell migration was observed by microscopy and analyzed by Fiji.

### Cell invasion assay

Cell invasion was assessed using a transwell with an 8 μm-pore of polycarbonate membranes coated by Matrigel matrix (Corning Inc., USA). Briefly, the cells (5 × 10^4^) were suspended in 100 μl of serum-free media and were then added to the upper chamber. Six hundred microliter of DMEM media were added to the lower chamber with 10% FBS. After 24 h, the invading cells were fixed with 4% formaldehyde and were stained with 0.1% crystal violet. The images were taken by microscope and the invading cells were counted.

### Colony formation assay

Cells were plated in 6-well plates at a density of 500 cells per well, then were subjected with the corresponding treatments followed by incubation for 10 days. The colonies formed were stained with 0.1% crystal violet and the visible colonies (more than 50 cells) were counted under a light microscopy.

### Cell proliferation analysis by BrdU incorporation

When the cells reached about 80% confluence in the 6-well plates, BrdU was added to the culture media at a final concentration of 30 μM BrdU. It was then incubated for 30 min at 37 °C. The cells were fixed, acid-treated, and detected using FITC-conjugated anti-BrdU antibody (eBioscience, 11-5071-42). The DNA was stained with 20 μg/mL of propidium iodide (PI). All samples were analyzed using a FACS Calibur flow cytometer (BD Biosciences) and FlowJo software.

### Detection of cell death by Annexin V/PI staining

Cells were seeded in 6-well culture plates and were induced to apoptosis by a treatment of tunicamycin for 48 h or gemcitabine for 72 h. The floating and adherent cells were collected, then co-stained with Alexa Fluor 488- conjugated Annexin V (Invitrogen) and PI. Samples were run within 30 min using flow cytometry. FlowJo software was used to analyze the data.

### Cell surface expression of epidermal growth factor receptor (EGFR)

Cells cultured in the 6-well plates were treated with 20 μM of gemcitabine for 48 h, washed with PBS and collected. The cells were then washed with PBS, fixed with 4% paraformaldehyde at room temperature for 10 min, and stained with Alexa Fluor 647-conjugated mouse anti-human EGFR (BD Biosciences, 563577) at 4 °C for 30 min. Samples were analyzed with flow cytometer and FlowJo software.

### Statistical analysis

All data analysis was conducted using SPSS 20.0 software. One-way analysis of variance (ANOVA) with Dunnett’s post-hoc test was used to analyze the differences between control cells and either AGR2-overexpressed cells. The data were expressed as means of three independent experiments ± the standard deviation (SD), with *p* < 0.05 considered statistically significant.

## Results

### AGR2 is localized in ER and KTEL motif deletion (△KTEL) of AGR2 is secreted

**Fig. 1:**
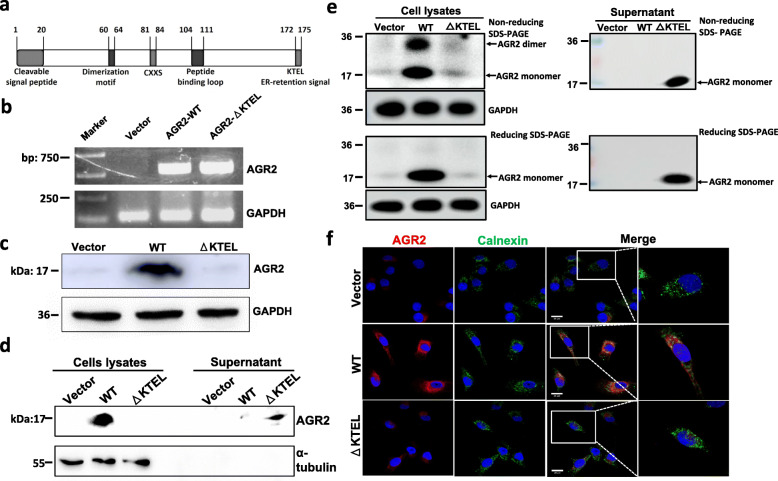
Generation of PANC-1 cells stably expressing ER-resident and secreted AGR2. **a** The primary structure of the AGR2 protein shows identified functional domains and motifs. **b** Expression levels of AGR2 mRNA in PANC-1 stable cell lines were detected by RT-PCR. **c** Western blotting was used to analyze the presentation of the AGR2 protein in whole cell lysates of PANC-1 stable cell lines. GAPDH served as a loading control. **d** The presence of AGR2 proteins in cell lysates and culture supernatant was detected by Western blotting. **e** DSP-stabilized AGR2 in cell lysates and culture supernatant was analyzed under non-reducing (top blot) or reducing conditions (bottom blot). **f** Cellular distribution of AGR2 and ER-associated protein, calnexin, were determined by immunofluorescence. AGR2 is shown in red, calnexin in green, and Hoechst 33342 in blue. Scale bars: 20 μm

Compelling evidences indicated that C-terminal KTEL motif was essential for AGR2 cellular localization (Fig. 1a) while deletion of the KTEL motif caused AGR2 secretion [11, 35–37]. To explore the functional significance of AGR2 cellular localization in pancreatic cancer cells, a lentivirus-mediated stable overexpression system was used (modified pLenti6.3 construct). PANC-1 cells with no detectable AGR2 were used to create control (Vector), wild type AGR2 (WT) and KTEL-deleted AGR2 (△KTEL) sublines. As expected, the mRNA expression of AGR2 markedly increased in the AGR2-WT and AGR2-△KTEL cell lines (Fig. 1b, sFig. 1). The protein expression of wild type AGR2 was observed in the whole cell extracts (Fig. 1c, sFig. 2), but not the culture supernatant (Fig. 1d, sFig. 3). Not only that, KTEL-deleted AGR2 was also only detected in the conditioned media (Fig. 1c and d, sFig. 2 and 3), suggesting that the loss of a KTEL motif results in AGR2 secretion. A recent report indicated that AGR2 exists in dimeric and monomeric status, which is regulated by the cellular regulators [34]. Consistent with this, intracellular AGR2 existed under both homodimeric and monomeric forms, while secreted AGR2 existed predominantly as homodimers (Fig. 1e, sFig. 4). To further investigate the cellular localization of AGR2, AGR2 was co-stained with calnexin, an ER internal protein marker. Consistent with the Western blotting assay, immunofluorescent staining in the PANC-1 stable cells indicated that wild type AGR2 was present in the cytoplasm and predominantly showed perinuclear distribution (Fig. 1f). Moreover, cellular AGR2 localization required a KTEL motif (Fig. 1f). Additionally, wild type AGR2 was observed to colocalize extensively with calnexin in PANC-1 cells (Fig. 1f), which further validated that intracellular AGR2 is an ER-resident protein. Taken together, AGR2 is localized in the ER, and the loss of a KTEL motif causes AGR2 to be secreted.

### Secreted AGR2, but not ER-resident AGR2, promotes cell migration, invasion, and proliferation

**Fig. 2:**
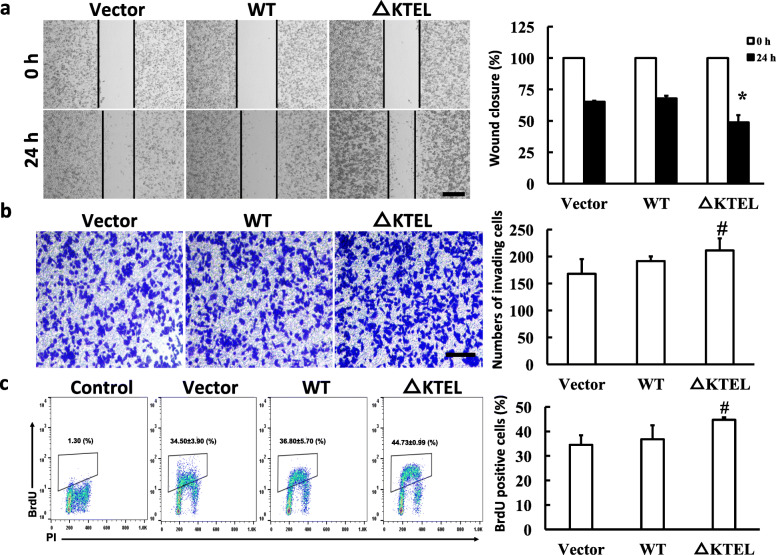
Extracellular AGR2, but not intracellular AGR2, promotes cell migration, invasion, and proliferation. **a** Wound healing assay was performed to measure the activity of cell migration in PANC-1 cells. The migration activities were calculated by relative wound. Scale bars: 1000 μm. **b** The invasion of the PANC-1 cells was evaluated by a transwell invasion assay. Right panel, the invading cells were counted. Scale bars: 500 μm. **c** Cell proliferation was detected by a BrdU incorporation assay, and the ratio of BrdU positive cells to the total cells was quantified by FlowJo software. *, *p* < 0.01 vs. Vector; #, *p* < 0.05 vs. Vector

**Fig. 3:**
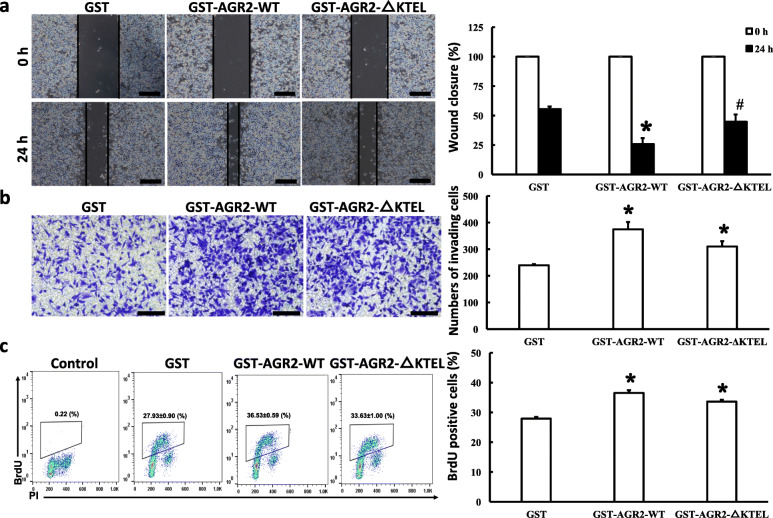
Recombinant AGR2 proteins promote PANC-1 cells migration, invasion, and proliferation in vitro. PANC-1 cells were seeded as described in Fig. 2 and treated with 200 nM of recombinant proteins of AGR2-WT, AGR2-△KTEL or GST as control for 24 h. And then the cell activities of migration (**a**), invasion (**b**) and proliferation (**c**) were detected as described in Fig. 2. Scale bars: 1000 μm in (**a**) and 500 μm in (**b**). *, *p* < 0.01 vs. Vector; #, *p* < 0.05 vs. Vector

AGR2 has been shown to facilitate cancer metastasis and growth in vitro and in vivo [4–10, 12, 38, 39]. However, the contribution of different localizations of AGR2 to pancreatic cancer has yet to be elucidated. Therefore, the effects of intracellular and extracellular AGR2 on cell migration, invasion, and proliferation in PANC-1 cells were assessed. Wound-healing assays indicated that PANC-1 AGR2-△KTEL cells had an increased migratory capacity compared to the control (Fig. 2a). Furthermore, an overexpression of secreted AGR2 also enhanced invasive capacity when compared to the control (Fig. 2b). BrdU assay was used to accurately assess the role of AGR2 in cell proliferation. As shown in Fig. 2c, AGR2-△KTEL cells displayed a remarkable increase in the number of cells in S phase compared to the control (44.73 ± 0.99% in S phase in AGR2-△KTEL cells vs 34.50 ± 3.90% in S phase in control cells), indicating that secreted AGR2 enhances cell proliferation in PANC-1 cells. Intriguingly, an overexpression of wild type AGR2 did not influence the migration, invasion, and proliferation of PANC-1 cells (Fig. 2a-c). To further investigate the secreted AGR2 function, GST-tagged AGR2-WT and AGR2-△KTEL were purified and added in the medium of PANC-1 cells (sFig. 5). Interestingly, both recombinant AGR2-WT and -△KTEL proteins promoted PANC-1 cells migration, invasion, and proliferation when compared with GST control (Fig. 3a-c), suggesting KTEL motif is necessary for cellular localization of AGR2, but is not essential for secreted AGR2 effects on tumorigenic properties. Taken together, these results demonstrate that secreted AGR2, but not intracellular AGR2, is associated with cell migration, invasion, and proliferation of PANC-1 pancreatic cancer cells under physiological conditions.

### AGR2 is required for the maintenance of ER homeostasis in two manners

**Fig. 4:**
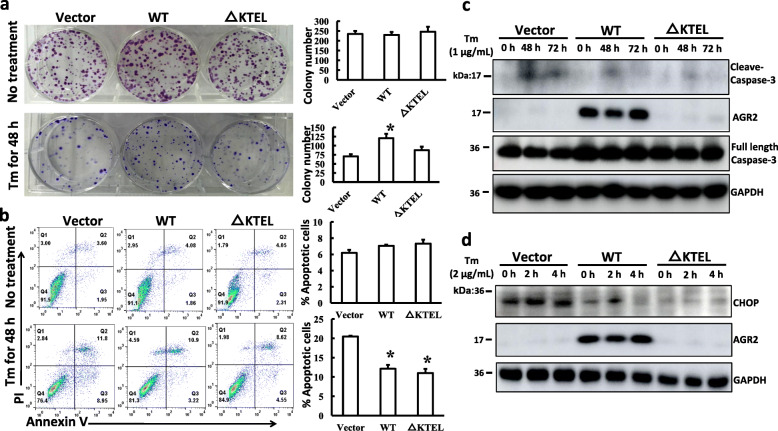
AGR2 promotes PANC-1 cell survival under ER stress induced by tunicamycin (Tm). **a** Colony assay was used to determine cell survival in the absence or presence of tunicamycin (1 μg/mL). **b** Tunicamycin-induced cell apoptosis was assessed by flow cytometry with the co-staining of PI and Annexin V. *, *p* < 0.01 vs. Vector. **c** PANC-1 stable cells were treated with tunicamycin (1 μg/mL) for the indicated times, and whole cell lysates were analyzed by Western blotting against cleaved Caspase-3 with GAPDH as loading control. **d** PANC-1 stable cells were treated with tunicamycin (2 μg/mL) for the indicated times, and the levels of CHOP were detected by Western blotting. GAPDH levels were measured as loading control

Considering that AGR2 is a member of the ER-resident PDI family and is involved in the control of ER homeostasis, the effect of intracellular and extracellular AGR2 on cell survival under ER stress was detected next. Overexpression of AGR2-WT and AGR2-△KTEL in PANC-1 cells had no obvious difference in the colony numbers and the apoptosis rate when compared with vector cells under an unperturbed condition (Fig. 4a-b). However, after tunicamycin (Tm)-induced ER stress, the colony-forming efficiency of the PANC-1 AGR2-WT cells was significantly enhanced compared to the control. The AGR2-△KTEL cells also marginally increased (Fig. 4a). Moreover, the Annexin V positive population decreased in both the AGR2-WT and AGR2-△KTEL cells compared to the control cells after tunicamycin treatment (Fig. 4b). Consistent with flow cytometry data, decreased levels of cleaved Caspase-3 were observed in both AGR2 overexpressed PANC-1 cells compared to control cells following tunicamycin treatment (Fig. 4c, sFig. 6). These data indicate that both intracellular and extracellular AGR2 can protect PANC-1 cells from ER stress-induced apoptosis. To further investigate the effects of AGR2 on ER homeostasis, an ER stress marker C/EBP homologous protein (CHOP) was detected in response to tunicamycin treatment. As shown in Fig. 4d and sFig. 7, overexpression of both intracellular and extracellular AGR2 significantly reduced CHOP expression under basal and tunicamycin-induced stress conditions. The above results demonstrated that AGR2 maintains ER homeostasis dependent of intracellular and extracellular manners.

### AGR2 expression modulates gemcitabine sensitivity

**Fig. 5:**
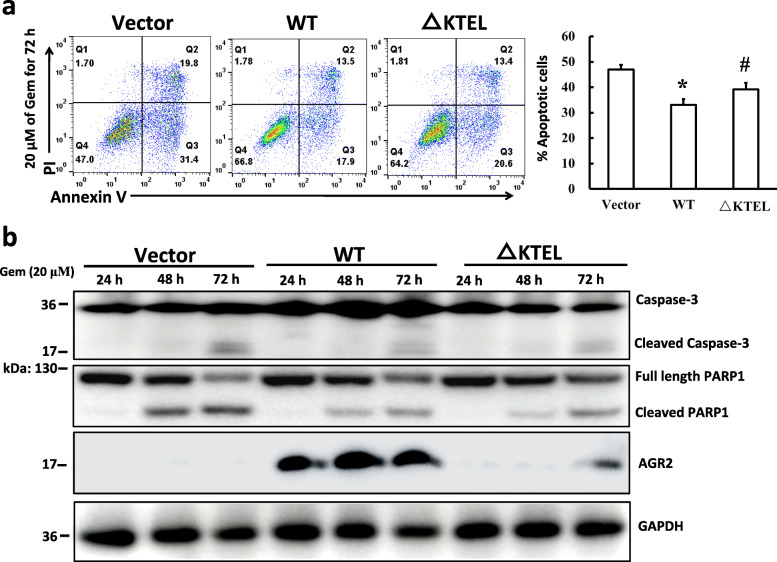
AGR2 expression modulates gemcitabine sensitivity of PANC-1 cells. **a** Indicative PANC-1 cells were treated with 20 μM gemcitabine (Gem) for 72 h, stained with Annexin V and PI, and then analyzed with flow cytometry. The results were analyzed with FlowJo software. *, *p* < 0.01 vs. Vector; #, *p* < 0.05 vs. Vector. **b** PANC-1 cells stably expressing the Vector or AGR2 were treated with 20 μM of gemcitabine (Gem) for the indicated periods of time. Whole cell lysates were collected and subjected to Western blotting against Caspase-3 and PARP1 with GAPDH as loading control

It has been widely reported that AGR2 is involved in the modulation of drug sensitivity in several cancers. Both the PANC-1 cells expressing intracellular and extracellular AGR2 displayed a decrease in the number of Annexin V positive cells after gemcitabine treatment when compared to the control (Fig. 5a), suggesting that either extracellular or intracellular AGR2 plays an important role in gemcitabine-induced cell apoptosis. Consistent with the flow cytometry data, reduced accumulations of cleaved Caspase-3 and PARP1 in both of PANC-1 cells expressing ER-resident and secreted AGR2 following gemcitabine treatment were noted (Fig. 5b, sFig. 8). Interestingly, intracellular and secreted AGR2 were dramatically induced upon gemcitabine treatment respectively in the PANC-1 AGR2-WT and -△KTEL cells (Fig. 5b, sFig. 8, 9), suggesting that increased AGR2 in turn promoted PANC-1 cells survival. These data prove that AGR2 contributes to chemotherapy sensitivity in PANC-1 pancreatic cancer cells in both ER-mediated and extracellular manners.

### AGR2 does not affect EGFR expression and activation

**Fig. 6:**
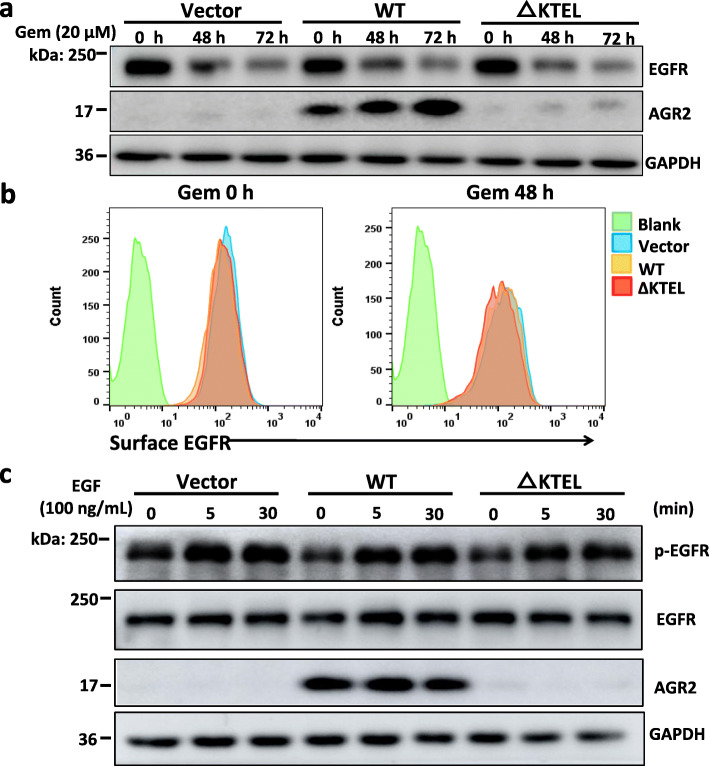
AGR2 does not affect EGFR expression and activation in PANC-1 cells. **a** The stable cells were treated with 20 μM gemcitabine (Gem) for the indicative times, and the levels of total EGFR protein were determined by Western blotting. **b** Flow cytometry was performed to assess levels of cell surface EGFR in untreated and Gem-treated cells. The signal intensity is plotted on the abscissa and the number of events on the ordinate. **c** The cells were treated with gemcitabine for the indicative times, and collected cell lysates were examined for the phosphorylated EGFR by Western blotting

AGR2 has been reported to regulate cell surface expression of EGFR by promoting receptor delivery from ER to cell surface [40]. Due to crucial roles of EGFR in growth, migration and cell survival, the levels of total and cell surface EGFR expression were analyzed in PANC-1 stable cells. As shown in Fig. 6a and sFig. 10, total EGFR protein was markedly decreased after gemcitabine treatment. However, no significant difference was observed in total EGFR levels between vector controls and either AGR2-overexpressed PANC-1 cells in both untreated and gemcitabine-treated conditions (Fig. 6a, sFig. 10). Importantly, AGR2 overexpression did not change the levels of cell surface EGFR in physiological and gemcitabine-treated conditions (Fig. 6b). Additionally, there was no significant change in the phosphorylation status of EGFR following EGF stimulation between control cells and either AGR2-overexpressed cells (Fig. 6c, sFig. 11). These data indicate that EGFR expression and activation are not involved in AGR-dependent oncogenic phenotypes in present cellular models.

## Discussion

In this study, PANC-1 cells stably expressing the ER-resident or secreted AGR2 were generated based on their biochemical structural characteristics. Secreted AGR2, but not ER-resident intracellular AGR2, was found to promote cell migration, invasion, and proliferation. Also, both intracellular and extracellular AGR2 were found to contribute to the survival of PANC-1 cells after tunicamycin-induced ER stress and gemcitabine-induced cell apoptosis. This demonstrates that two different AGR2-mediated pathways are possibly involved in the ER stress response and drug sensitivity.

Accumulating evidence suggests that AGR2 is an ER-resident protein with PDI activity and is required for the maintenance of ER homeostasis. Three key linear motifs in its primary structure are required for its biological function and ER localization. Firstly, the human AGR2 protein has a putative secretory signal peptide sequence, which includes the first 20 amino acid residues in its N-terminus (Fig. 1a), that directs AGR2 into the ER [14]. Secondly, an ER retention-like motif at the C-terminal of an AGR2 protein, also known as a tetrapeptide of KTEL, is able to bind to canonical KDEL receptors for ER localization (Fig. 1a) [35]. The deletion of the KTEL motif in different types of cell lines results in AGR2 secretion [35–37]. This study showed that wild type AGR2 localizes in the ER of PANC-1 cells, while AGR2 lacking the KTEL motif is secreted into culture media. These findings support the notion that the KTEL motif is a crucial biochemical determinant for the cellular localizations of AGR2 in pancreatic cancer cells. Another key motif is the CXXS thioredoxin (TX) activity motif, which is able to form mixed disulfide bonds with substrate proteins and is involved in ER protein folding, maturation, and secretion (Fig. 1a) [3, 40–42]. A previous study reported that AGR2 forms a mixed disulfide with EGFR and regulates the cell surface EGFR expression by promoting receptor delivery from ER to the plasma membrane, which is required for EGFR-mediated signal transduction [40]. The impact of AGR2 on EGFR presentation to the cell surface is essential for pancreatitis-associated tissue regeneration in mice [43]. However, the overexpression of ER-resident and secreted AGR2 does not change the cell surface expression and activation of EGFR in PANC-1 cells under physiological and gemcitabine-treated conditions, suggesting that the effects of AGR2 on cell proliferation, migration, invasion and survival are EGFR-independent. One possible explanation for the discrepancy in EGFR expression at the cell surface is that there were differences in cell lines and AGR2’s expression patterns (overexpression versus knockdown or knockout) used in the different studies.

In addition to EGFR expression, intracellular AGR2 is associated with the upregulation of multiple pro-survival and pro-metastatic molecules, such as cathepsin B (CTSB), CTSD, cyclin D1, survivin, mucin, amphiregulin (AREG) and transcription factor CDX2 [6, 7, 33, 35]. In the present study, intracellular AGR2 attenuates ER stress and gemcitabine-induced cell death, but it does not impact oncogenic phenotypes under physiological condition, suggesting ER-resident AGR2 predominantly contributes to cell survival during ER stress and chemotherapy.

Notably, AGR2 can be found in the extracellular matrix of cultured cells and the serum or urine of cancer patients [4, 8, 15, 16, 20, 21]. Stably silencing of AGR2 in MPanc-96 pancreatic cancer cells in which AGR2 is present in both cell lysates and conditioned media reduces cell proliferation and invasion [4]. Importantly, a recent study reported that overexpression of extracellular AGR2 in MiaPaCa-2 pancreatic cancer cells promotes tumor metastasis in vivo models [37]. Additionally, it has also been reported that the administration of recombinant AGR2 promotes growth, migration, invasion of cancer cells [36, 37, 39, 44]. Furthermore, AGR2vH, a spliced variant of AGR2 lacking the C-terminal KTEL motif, dramatically promotes cancer cell migration and invasion in vitro [45]. Together with our findings that secreted AGR2 promotes PANC-1 cell migration, invasion, and proliferation, these data suggest that extracellular AGR2 plays a key role in pancreatic cancer initiation and progression. Mechanistically, AGR2 interacts with C4.4A, a cell surface receptor of the Lys superfamily. The AGR2-C4.4A pathway is involved in the growth and metastasis of pancreatic tumors [46]. Moreover, extracellular AGR2 was observed to directly interact with and enhance the vascular endothelial growth factor (VEGF) and fibroblast growth factor 2 (FGF2), contributing to angiogenesis and tumor growth [44]. In addition, some studies have shown that secreted AGR2 helps regulate Wnt, mTORC and Hippo signal pathways [37, 39]. Interestingly, extracellular and intracellular AGR2 have the opposing effects on the mTORC and Hippo pathways [37]. However, the underlying mechanism of this process has not yet been established. Therefore, further studies discussing the mechanism of secreted AGR2 in tumor growth and metastasis are necessary.

Our data also demonstrates that wild type AGR2 localizes in the ER, while AGR2 lacking the KTEL motif is secreted into extracellular media. Moreover, both ER-resident and secreted AGR2 contribute to the control of ER homeostasis and gemcitabine sensitivity, which is consistent with a previous study in which AGR2 silencing enhances gemcitabine sensitivity [4]. This indicates that AGR2-mediated cell survival involves two pathways: ER-mediated and extracellular. A previous report showed that several distinct splice variants of AGR2 were present in cancer lines, tissue biopsies, and urine exosomes [47]. Additionally, AGR2 was detected in both the cell lysates and conditioned media in some cancer cell lines, or in both the carcinoma biopsies and urine exosomes of prostate cancer patients [4, 47]. Collectively, these findings imply that multiple splice variants of AGR2 may be present in the same cancer cell lines or in the same cancer patients, and that ER-resident and secreted AGR2 may simultaneously be involved in tumor development and chemotherapeutic sensitivity.

## Conclusions

In conclusion, the results of the present study indicate that extracellular AGR2 markedly promotes cell migration, invasion, and proliferation in PANC-1 pancreatic cancer cells. Both ER-resident and secreted AGR2 are involved in PANC-1 cells survival after tunicamycin-induced ER stress and gemcitabine treatment. Moreover, EGFR expression and activation are not involved in AGR2 pro-oncogenic roles in PANC-1 pancreatic cancer cells. These data extend our understanding of AGR2’s different localizations and their roles in pancreatic cancer growth, metastasis, and drug sensitivity.

## Supplementary Information

**Additional file 1.**

**Additional file 2.**

**Additional file 3.**

**Additional file 4.**

**Additional file 5.**

**Additional file 6.**

**Additional file 7.**

**Additional file 8.**

**Additional file 9.**

**Additional file 10.**

**Additional file 11.**

## Data Availability

All the data generated and/or analyzed during this study are included in this published article and will be available from the corresponding author on reasonable request.

## Abbreviations

AGR2: Anterior gradient-2
ER: Endoplasmic reticulum
EGFR: Epidermal growth factor receptor
GST: Glutathione S-transferase
PDI: Protein disulfide isomerase
WT: Wild type
PCR: Polymerase chain reaction
GAPDH: Glyceraldehyde 3-phosphate dehydrogenase
BrdU: 5-bromo-2′-deoxyuridine
Gem: Gemcitabine
Tm: Tunicamycin
