# Supplementary figures and images for: Effects of ER-resident and secreted AGR2 on cell proliferation, migration, invasion, and survival in PANC-1 pancreatic cancer cells
Source: BMC Cancer. 2021 Jan 7;21:33. doi: 10.1186/s12885-020-07743-y (PMC7791724; doi:10.1186/s12885-020-07743-y)

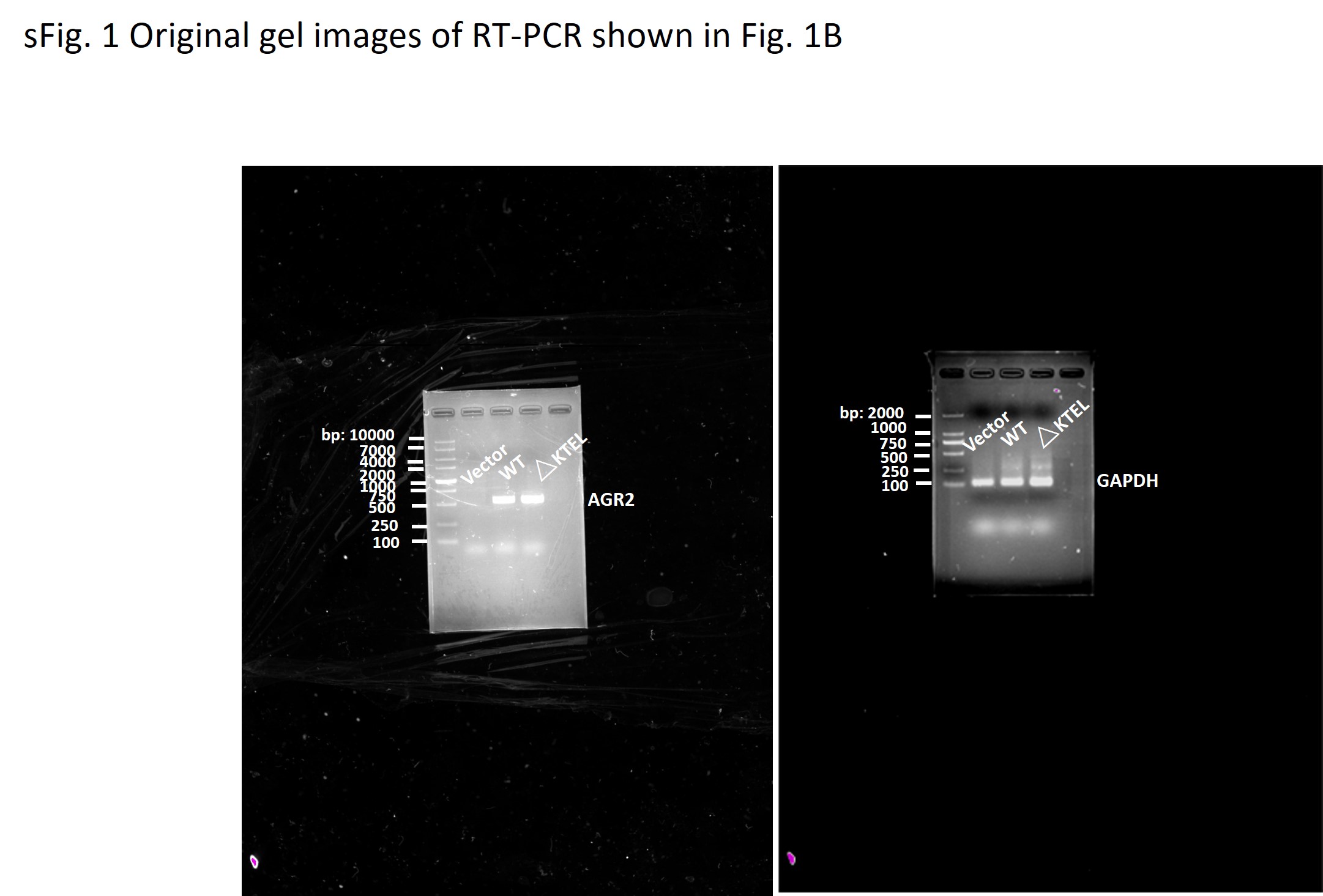

Supplement: Supplementary file 1 — Additional file 1. [file 12885_2020_7743_MOESM1_ESM.jpg]

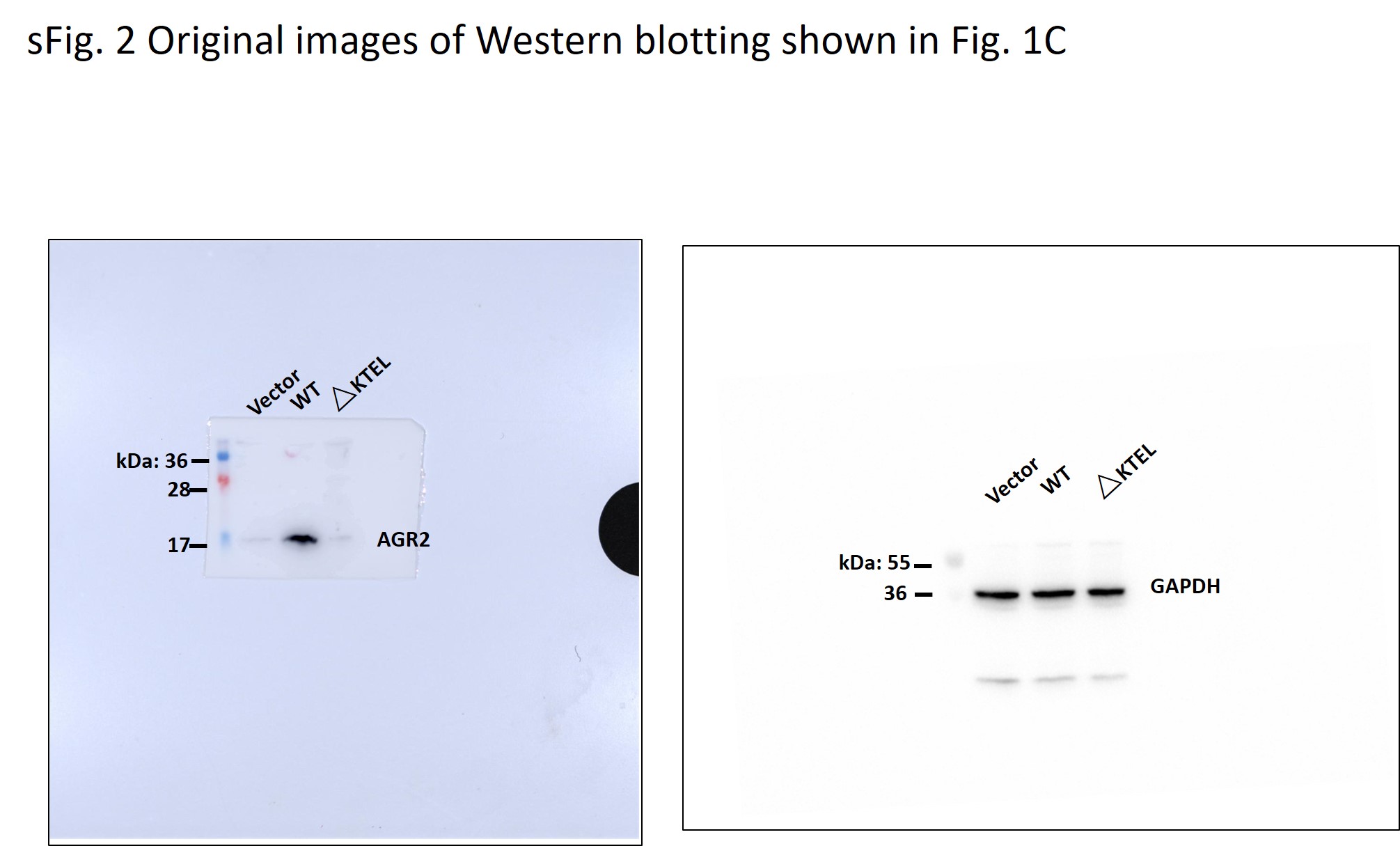

Supplement: Supplementary file 2 — Additional file 2. [file 12885_2020_7743_MOESM2_ESM.jpg]

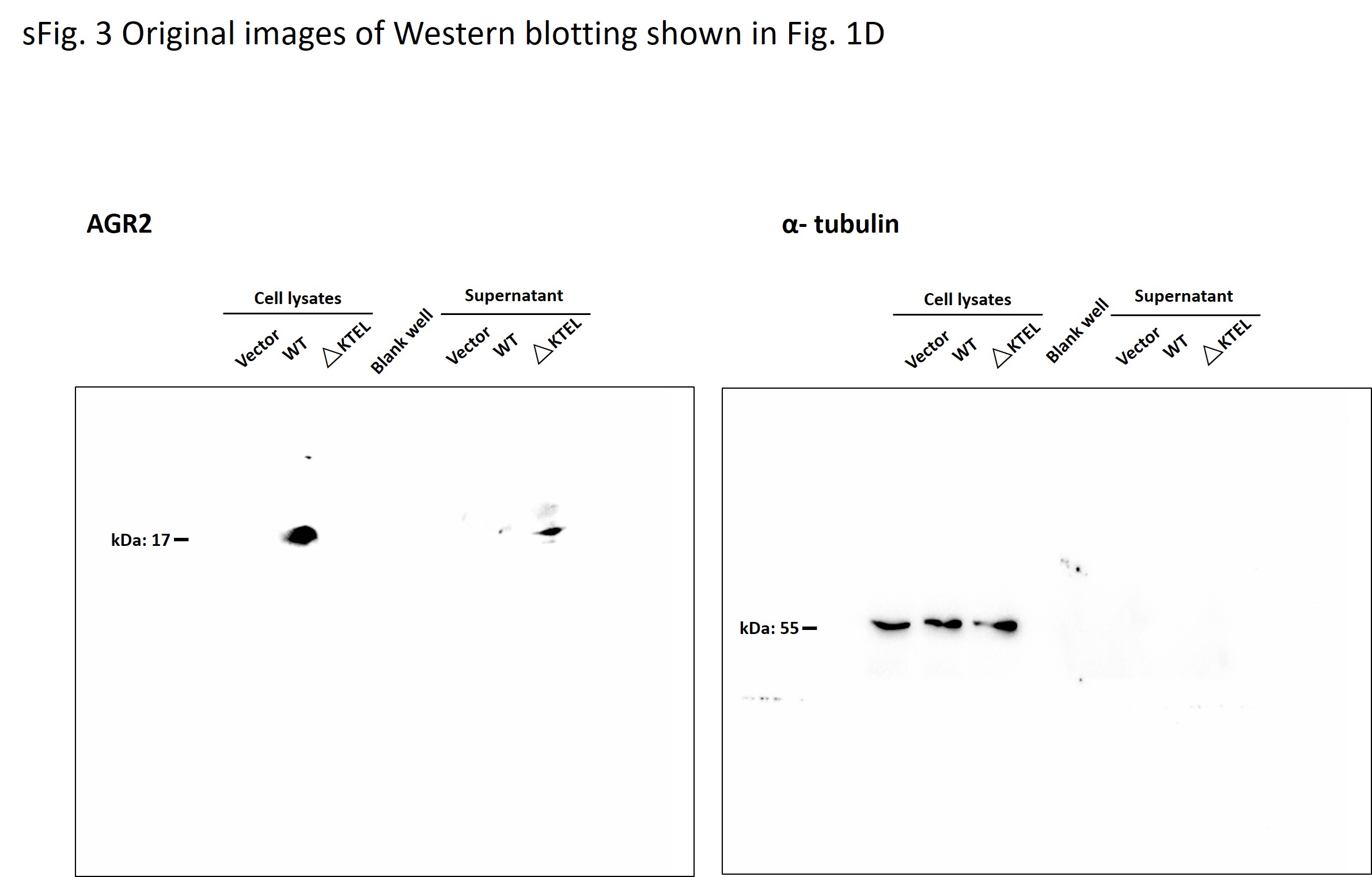

Supplement: Supplementary file 3 — Additional file 3. [file 12885_2020_7743_MOESM3_ESM.jpg]

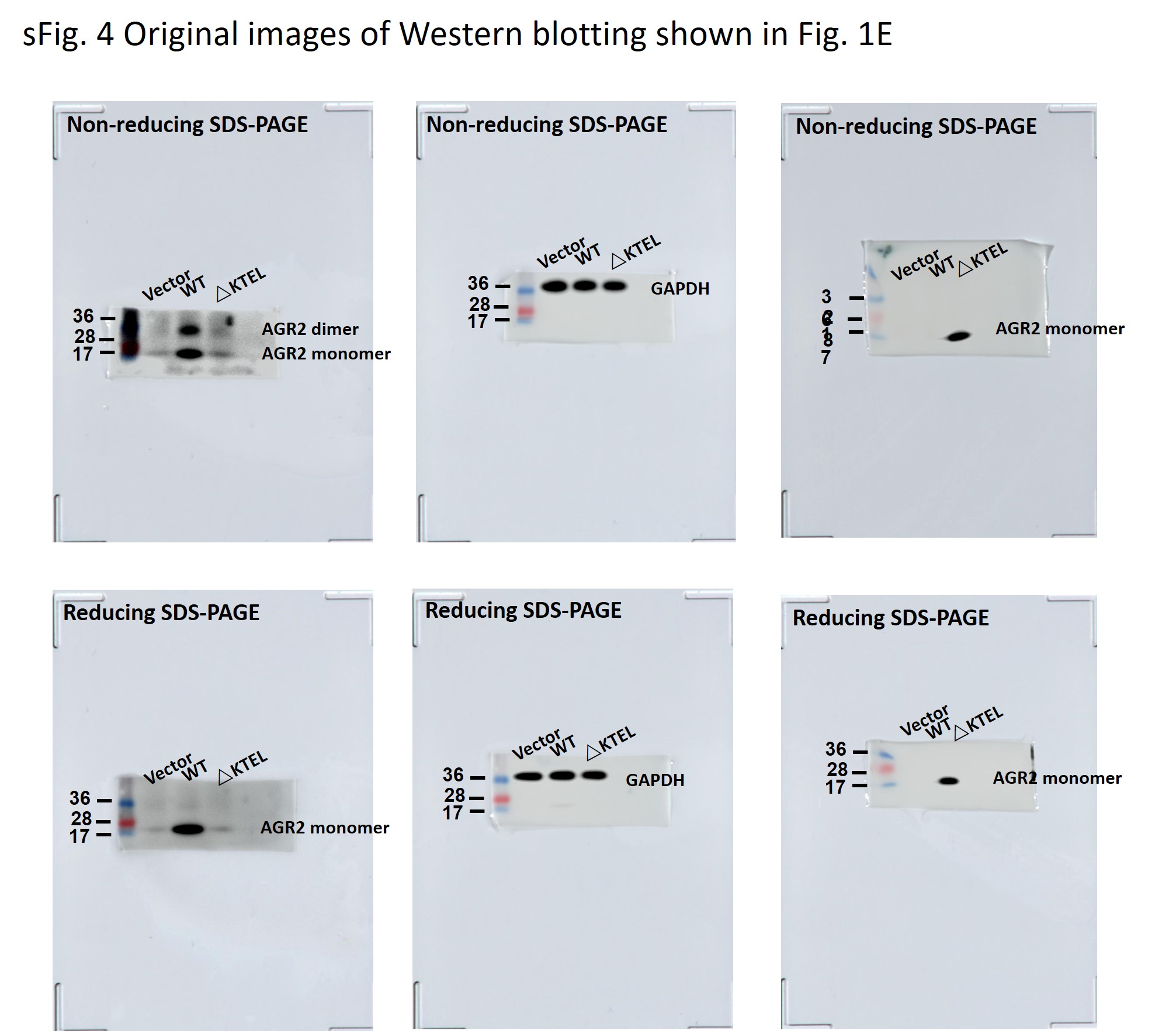

Supplement: Supplementary file 4 — Additional file 4. [file 12885_2020_7743_MOESM4_ESM.jpg]

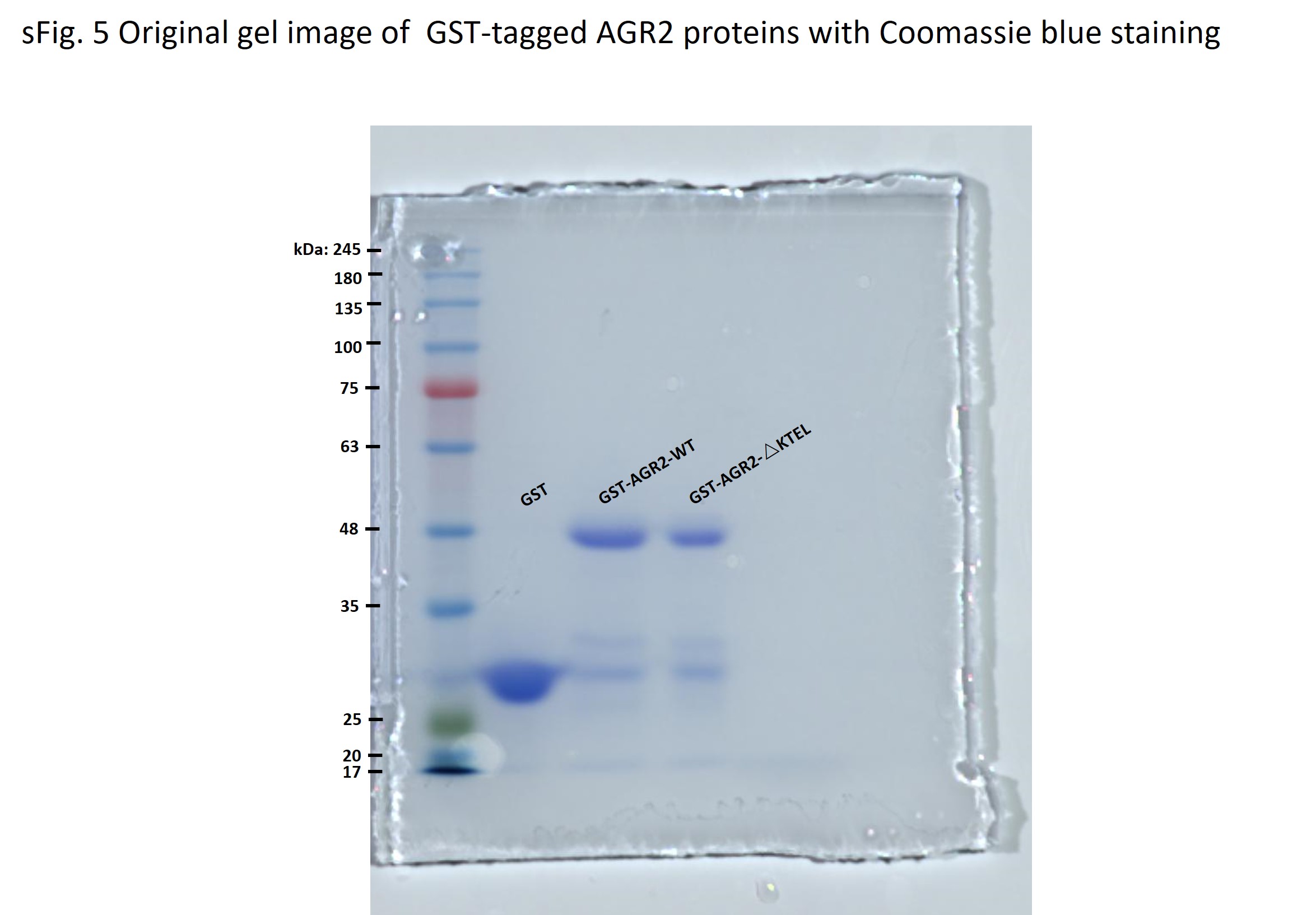

Supplement: Supplementary file 5 — Additional file 5. [file 12885_2020_7743_MOESM5_ESM.jpg]

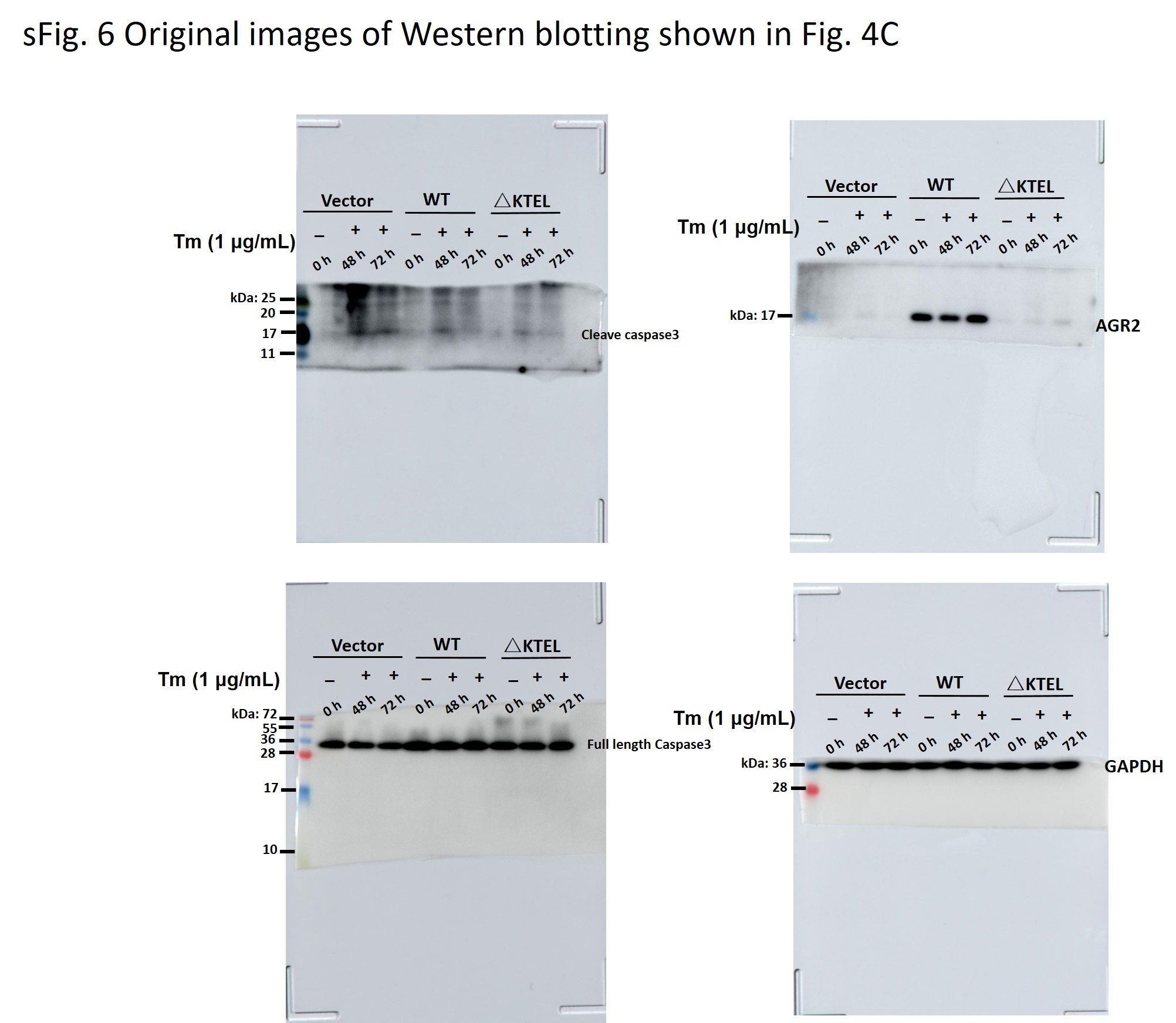

Supplement: Supplementary file 6 — Additional file 6. [file 12885_2020_7743_MOESM6_ESM.jpg]

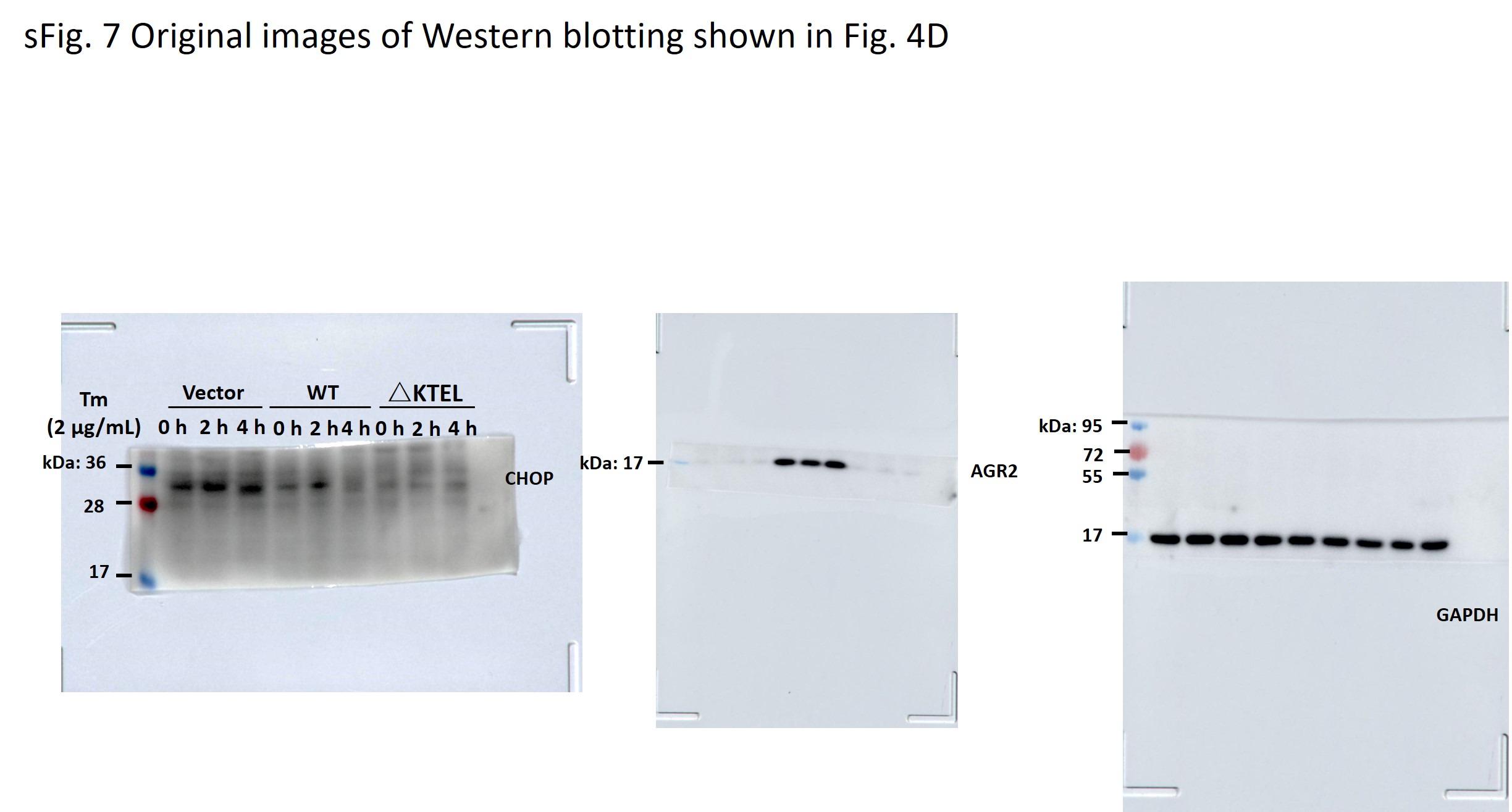

Supplement: Supplementary file 7 — Additional file 7. [file 12885_2020_7743_MOESM7_ESM.jpg]

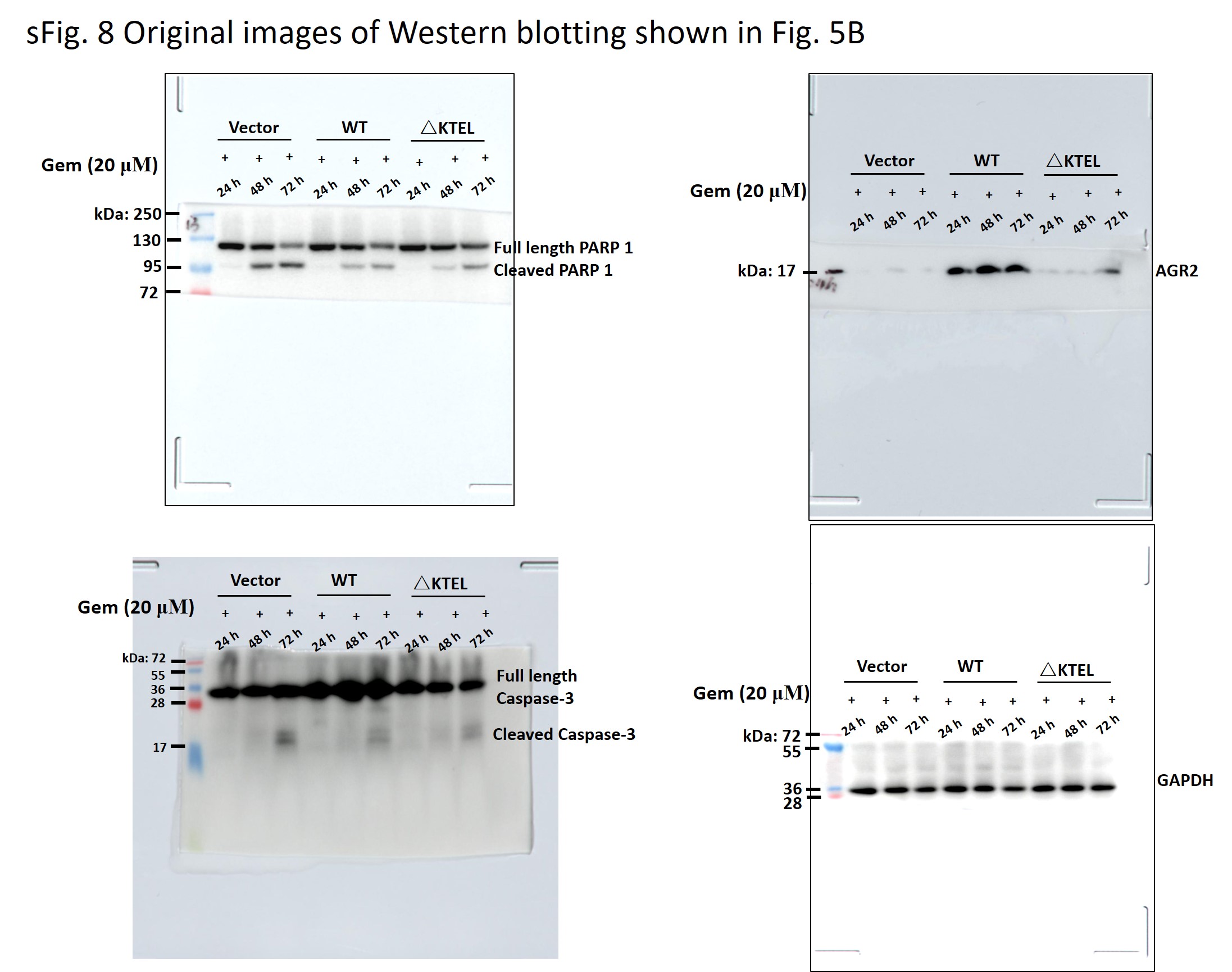

Supplement: Supplementary file 8 — Additional file 8. [file 12885_2020_7743_MOESM8_ESM.jpg]

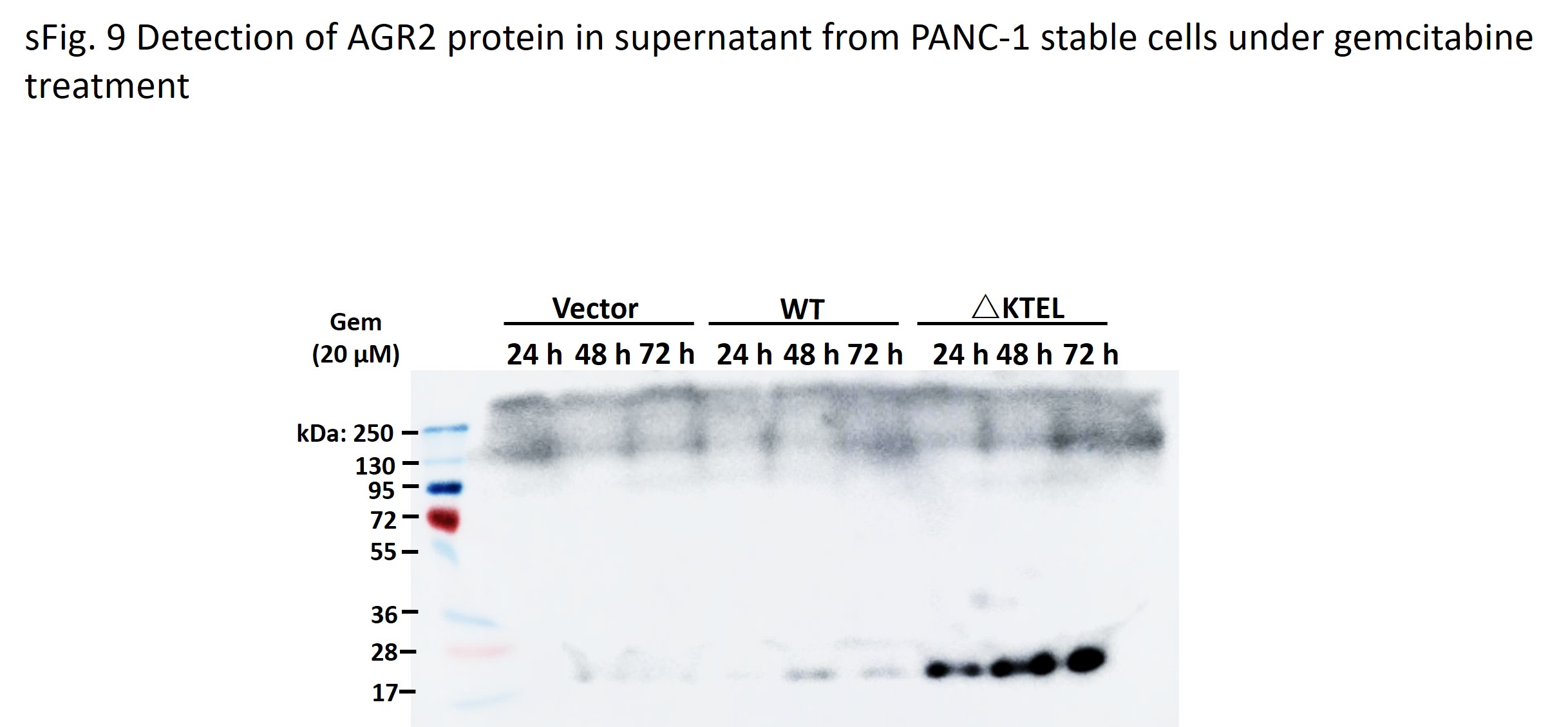

Supplement: Supplementary file 9 — Additional file 9. [file 12885_2020_7743_MOESM9_ESM.jpg]

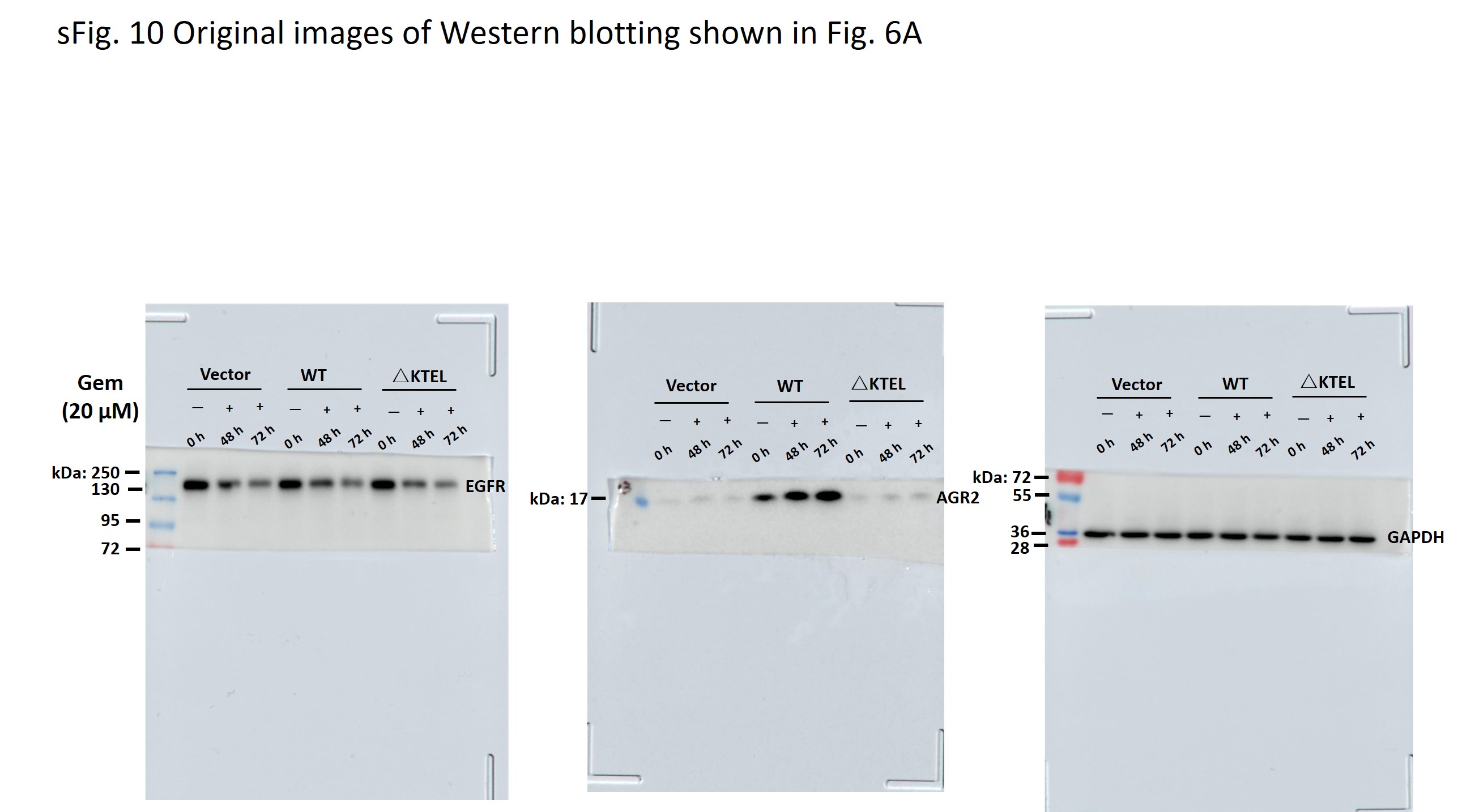

Supplement: Supplementary file 10 — Additional file 10. [file 12885_2020_7743_MOESM10_ESM.jpg]

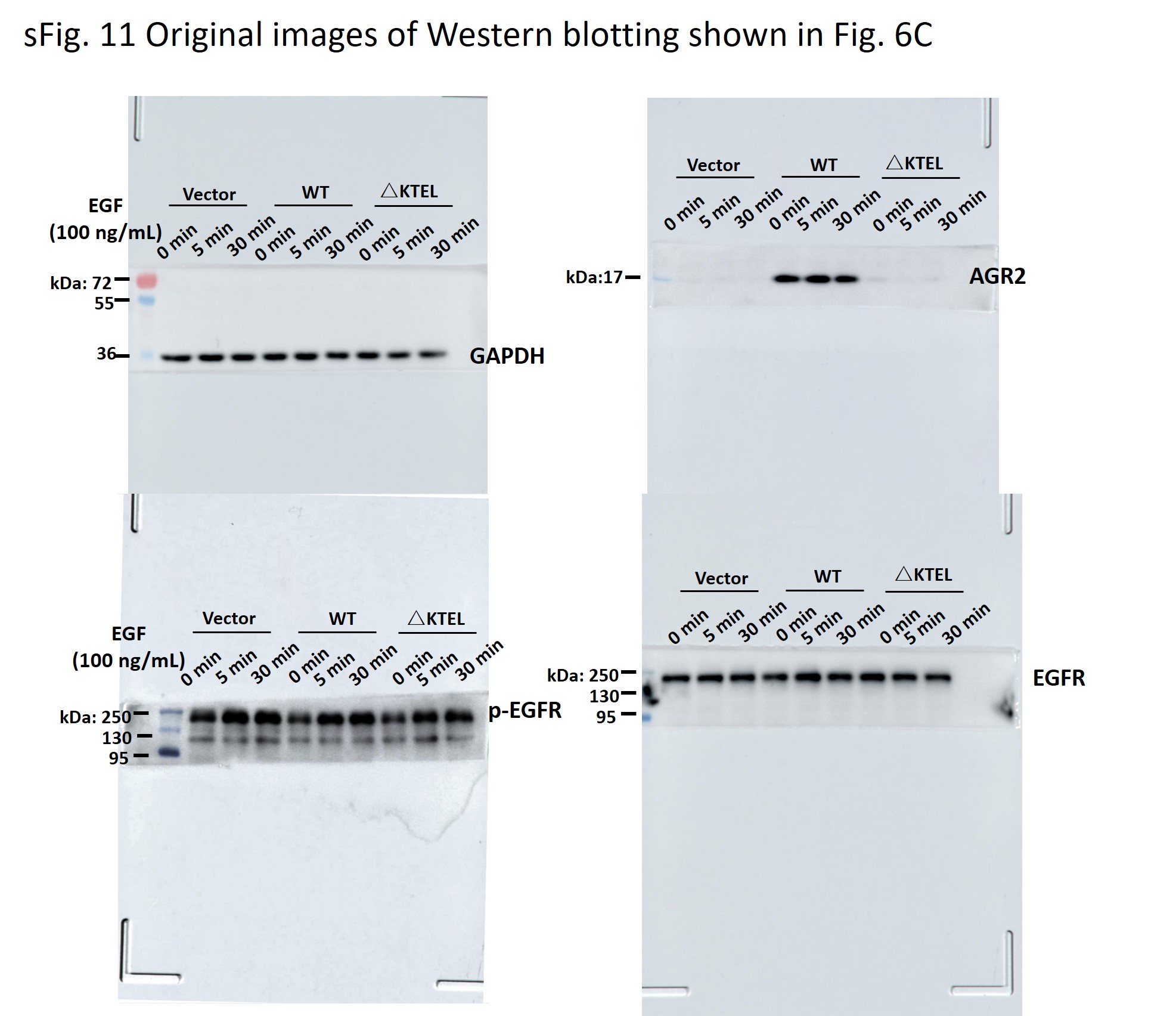

Supplement: Supplementary file 11 — Additional file 11. [file 12885_2020_7743_MOESM11_ESM.jpg]
